# Assessment of nutrition, quality of life, and co-morbidity among the older adult(s), in a rural field practice area of a tertiary care hospital of Eastern India

**DOI:** 10.3389/fpubh.2026.1851457

**Published:** 2026-10-05

**Authors:** Rosy Nayak, Smrutiranjan Nayak, Prem Sagar Panda, Ipsita Debata, Subhadra Priyadarshini

**Affiliations:** Department of Community Medicine, Kalinga Institute of Medical Sciences, Bhubaneswar, Odisha, India

**Keywords:** comorbidity, older population health, malnutrition, Mini Nutritional Assessment (MNA), quality of life, rural population, WHOQOL-BREF

## Abstract

**Background:**

The global population is experiencing an unprecedented demographic shift, with a growing proportion of older adults, particularly in developing nations such as India. Nutritional deficiencies, comorbidities, and poor quality of life (QoL) are interlinked health issues that significantly affect the wellbeing of the older adult(s), a vulnerable group, especially those residing in rural areas.

**Objectives:**

To assess the nutritional status, quality of life, and comorbidities among the older population residing in the rural field practice area of a tertiary care hospital in Eastern India and to explore associations among these factors.

**Methods:**

A community-based, cross-sectional study was conducted among 380 rural older adult(s) aged 60 years and above residing in the rural field practice area of a tertiary care hospital in Bhubaneswar, Odisha. Data were collected using the Mini Nutritional Assessment (MNA), World Health Organization Quality of Life-BREF (WHOQOL-BREF), and Self-Administered Comorbidity Questionnaire (SCQ). Compiled data were analyzed using IBM SPSS Statistics for Windows, version 26.0 (IBM Corp., Armonk, NY). Descriptive data were interpreted in mean (± SD), frequencies, and percentages. Association was tested using the chi-square test, ANOVA, and multivariate logistic regression. Statistical significance was established at *p* < 0.05.

**Results:**

A total of 380 older adult(s) participants were enrolled (mean age: 71.23 ± 7.60 years, males: 65.8%). Around 4.2% (16) were malnourished, and 69.7% (265) were at risk of malnutrition. The WHOQOL-BREF scores revealed the highest QoL in the environmental domain (27 ± 5) and the lowest in the social relationship domain (8 ± 2). Using the SCQ tool, osteoarthritis (64.7%), hypertension (32.6%), and diabetes (24.2%) were the most common comorbidities, and 82.6% suffered from at least one comorbidity. Increasing age, female sex, and financial dependency were significantly associated with poor nutritional status and lower QoL (*p* < 0.05). Malnutrition was significantly associated with lower physical, psychological, and total QoL scores (*p* < 0.05).

**Conclusion:**

Malnutrition, comorbidities, and poor QoL are interrelated challenges among the older population. Early identification and integrated interventions focusing on nutrition, chronic disease management, and social support are vital for healthy aging.

## Introduction

India is currently experiencing a rapid demographic transition, with a steadily increasing proportion of older population in its population. As life expectancy continues to rise, there is a parallel increase in the burden of chronic diseases, nutritional deficiencies, and declining quality of life (QoL) among older adults. According to the World Health Organization (WHO), health is not merely the absence of disease but a state of complete physical, mental, and social wellbeing (1). This holistic definition emphasizes that promoting health among the older adult(s) requires attention beyond medical care alone; it demands focus on their psychological and social needs as well.

In rural India, older adults face unique and multifaceted challenges that exacerbate health inequalities. Limited access to healthcare facilities, financial insecurity, social isolation, and dependency on family members often combine to worsen their overall wellbeing. Among these, malnutrition remains a critical yet under-recognized issue, with national prevalence rates ranging between 13% and 54% (2). Inadequate dietary intake, functional decline, and the presence of chronic conditions such as hypertension, diabetes, and arthritis further heighten the risk of poor nutritional status. These interconnected factors create a self-reinforcing cycle: chronic disease impairs appetite and nutrient absorption, malnutrition accelerates functional decline, and both together drive a progressive deterioration in QoL that is especially severe in resource-limited rural settings. Quality of Life (QoL), as defined by the WHO, reflects an individual's perception of their position in life within the context of their culture, value system, goals, and expectations (3–5). Unlike traditional clinical measures that focus on disease and mortality, QoL encompasses broader dimensions of wellbeing, including psychological, social, and environmental factors. For the older population, this perspective is especially important, as physical disability, mental health issues, and economic dependency often lead to a significant decline in perceived QoL (6). Furthermore, the presence of multiple chronic illnesses (multi-morbidity) compounds these problems by limiting functional independence and reducing participation in daily activities (7). The Ministry of Health & Family Welfare reports that approximately 75% of Indians over 60 years have at least one chronic disease (8). In rural Eastern India specifically, healthcare access is further constrained by inadequate infrastructure, shortage of geriatric specialists, geographic remoteness, low health literacy, and dependence on informal caregivers—all of which compound the risk of undetected and unmanaged morbidity in this population. Access to medical personnel, diagnostic tools, and health education plays a significant role in the under-reporting and misdiagnosis of comorbid conditions among older populations in rural areas. Because many chronic illnesses remain undetected or inadequately documented, accurate estimation of morbidity burden becomes challenging. In such settings, standardized and validated comorbidity assessment tools are essential for generating reliable data, improving case detection, and guiding clinical as well as public health interventions. A variety of instruments have been developed to assess comorbidity and multi-morbidity, each differing in structure, scoring, and applicability. Among these, the Self-Administered Comorbidity Questionnaire (SCQ) has emerged as a practical and widely used tool, particularly in community-based studies and low-resource settings. The SCQ allows individuals to report their own chronic conditions, treatments, and functional limitations, thereby overcoming many of the barriers posed by poor health records, limited diagnostic services, and inadequate medical follow-up—barriers that are common in rural older populations. Given these challenges, it is crucial to understand the interplay between nutritional status, comorbidities, and quality of life among the older population. Such evidence can guide the design of targeted interventions and policies to promote healthy and active aging, particularly in rural communities where healthcare resources are limited.

Despite the growing recognition of these challenges, robust evidence linking malnutrition, multimorbidity, and QoL specifically among community-dwelling older adult(s) in rural Eastern India remains scarce. The present study addresses this evidence gap and aims to generate locally relevant data to inform geriatric healthcare policy and community-based interventions in low-resource settings.

## Materials and methods

### Study design, setting, duration

A community-based, cross-sectional study was conducted among the older adult(s), aged 60 years and above, residing in the rural field practice area of the Department of Community Medicine, Kalinga Institute of Medical Sciences (KIMS), KIIT University, Odisha, between January 2023 and March 2025.

### Inclusion and exclusion criteria

Older adult(s) aged 60 years and above, residing in the study area for at least 6 months, were included. Bedridden or terminally ill individuals were excluded.

### Sample size

The rural area encompasses 39 villages with a population of approximately 54,000 individuals. The sample size of 380 was calculated using the formula *n* = Z^2^PQ/d^2^, assuming a prevalence (*p*) of malnutrition of 31%, from a study by Sharma et al. (9), a 95% confidence level, and 5% absolute precision, the minimum required sample size was calculated as 329. A 10% non-response rate was added, yielding 329 + 33 = 362, which was rounded up to 380. Systematic random sampling was employed to select participants.

### Data collection tools

Data were collected using a pre-tested semi-structured questionnaire comprising four parts: (I) socio-demographic information, (II) Nutritional status was assessed by the Mini Nutritional Assessment (MNA) scale (3), (III) QoL by the WHOQOL-BREF (6) scale, and (IV) comorbidity status by the Self-Administered Comorbidity Questionnaire (SCQ) (7). The socioeconomic status was assessed using the Modified B.G. Prasad classification 2024 (10). As the data were collected from January 2023 to March 2025 and the B.G. Prasad scale's income cut-offs are revised annually for inflation, the per-capita family income/month of all participants was uniformly reclassified as per the 2024 CPI-IW-adjusted cut-offs at the time of data compilation, irrespective of the participant's actual month of recruitment. This was done to ensure a single, internally consistent classification across the study period. The MNA is a validated 18-item tool with a sensitivity of 96% and specificity of 98% for detecting malnutrition in community-dwelling older adult(s); it has been widely used in Indian geriatric studies (3). The WHOQOL-BREF is a 26-item WHO-validated instrument with established reliability (Cronbach's α ≥ 0.70 across all domains) and cross-cultural applicability (6). The SCQ is a validated self-report comorbidity tool with good test-retest reliability (intraclass correlation coefficient ≥0.84) (7).

### Sample collection technique

Field investigators were trained, and informed consent was obtained. The total number of households in the field practice area of the Rural Health Center was obtained from the register, after which systematic random sampling was employed to select the households. Households that were locked even after three visits were excluded from the study. A two-stage sampling technique was employed. In Stage 1, 10 wards were selected from the 39 villages by simple random sampling (lottery method). In Stage 2, within each selected ward, the sampling interval was calculated as the total number of eligible households divided by the required sample per ward. The starting household within each ward was identified using a random spin of a pencil. In cases where none of the household members was considered eligible, a participant from the adjacent household with the closest front door was approached. Participant replacement for locked/absent households followed a predefined protocol (adjacent household approach), ensuring that the replacement rate did not exceed 5% of the total sample. In cases where multiple eligible participants were in the same household, only one was randomly selected by lottery. Formal design effect calculation was not performed; however, the systematic within-ward approach was applied consistently across all selected wards. Anthropometric measurements, including height, weight, mid-arm, and calf circumferences, were taken using standardized procedures.

### Statistical analysis

Data were entered in Microsoft Excel 2016 version and analyzed using the SPSS software (IBM Corp., 2019, IBM SPSS Statistics for Windows, version 26.0, Armonk, NY: IBM Corp.). Continuous variables were expressed as mean ± SD, and categorical variables as frequencies and percentages. Normality of continuous variables was assessed using the Shapiro-Wilk test. Normally distributed variables were compared using ANOVA; non-normally distributed variables were analyzed using the Kruskal-Wallis test. Chi-square and Fisher's exact tests were used for categorical associations. Multivariable binary logistic regression was performed to identify factors independently associated with nutritional status. Variables with *p* < 0.20 in bivariate analysis were entered into the model using an enter method. Adjusted odds ratios (AOR) with 95% confidence intervals (CI) are reported. Model fit was assessed using the Hosmer-Lemeshow goodness-of-fit test, and multicollinearity was evaluated using the Variance Inflation Factor (VIF; threshold < 5). A *p*-value < 0.05 was considered statistically significant.

### Operational definitions

Quality of life was categorized as “low QOL” (total WHOQOL-BREF score ≤ 80) and “better QOL” (score >80) based on the median score of the study population, as no validated population-specific cut-off exists for rural Indian older population. Multimorbidity was defined as the presence of two or more concurrent chronic conditions identified by the SCQ. Financial dependency was defined as reliance on family members or others (not self) for medication expenditure, as captured in the sociodemographic questionnaire.

### Ethical consideration

Ethical clearance was obtained from the Institutional Ethics Committee, Kalinga Institute of Medical Sciences (Ref. No.: KIIT/KIMS/IEC/2078/2025), and the study was conducted in accordance with the Declaration of Helsinki. Informed written consent was taken from the participants, and confidentiality was assured. This study was reported in accordance with the STROBE (Strengthening the Reporting of Observational Studies in Epidemiology) guidelines for cross-sectional studies.

## Results

A total of 380 older adult(s) participants were included in the study. The majority of participants (64.7%) were aged 60–69 years, while 35.3% were aged ≥70 years. Most participants were male (65.8%), and 34.2% were female. Nearly all participants belonged to the Hindu religion (97.9%), with 2.1% Muslims. Regarding occupational status, 66.1% were employed, whereas 33.9% were unemployed. The majority of the participants were literate (77.9%), while 22.1% were illiterate.

Based on socioeconomic classification, 60.8% of the participants belonged to the lower socioeconomic class, followed by 30.5% in the lower middle class, 5.5% in the middle class, 1.3% in the upper middle class, and 1.8% in the upper class.

Overall, 82.6% (95% CI: 78.5–86.1%; 314/380) of participants reported at least one comorbidity, while 17.4% had no comorbid condition. Regarding monthly expenditure on medicines, 34.5% spent 1,000–5,000, 33.7% spent less than 1,000, 29.7% reported no expenditure, and 2.1% spent more than 5,000. Medication expenditure was self-financed by 41.3% of participants, supported by others in 32.1%, and 26.6% reported no medication expenditure.

Assessment of nutritional status using the Mini Nutritional Assessment (MNA) showed that 4.2% (95% CI: 2.6–6.7%; 16/380) of participants were malnourished, 69.7% (95% CI: 64.9–74.1%; 265/380) were at risk of malnutrition, and 26.1% (95% CI: 21.9–30.7%; 99/380) had satisfactory nutritional status, as depicted in Table 1.

**Table 1:** Socio-demographic characteristics, comorbidity status, and nutritional status of the study participants (*n* = 380).

Characteristics	Category	Frequency (%)
Age (in years)	60–69	246 (64.7)
	>=70	134 (35.3)
Gender	Male	250 (65.8)
	Female	130 (34.2)
Religion	Hindu	372 (97.9)
	Muslim	8 (2.1)
Occupational status	Unemployed	129 (33.9)
	Employed	251 (66.1)
Educational status	Literate	296 (77.9)
	Illiterate	84 (22.1)
Monthly income	Lower class	231 (60.8)
	Lower middle class	116 (30.5)
	Middle class	21 (5.5)
	Upper middle class	5 (1.3)
	Upper class	7 (1.8)
Comorbidity	No	66 (17.4)
	Yes	314 (82.6)
Monthly expenditure on medicine (Rupees)	Nil	113 (29.7)
	<1,000	128 (33.7)
	1,000–5,000	131 (34.5)
	>5,000	8 (2.1)
Sponsor of Medication expenditure	Nil	101 (26.6)
	Own	157 (41.3)
	Other	122 (32.1)
MNA score	Malnourished (MNA score < 17)	16 (4.2)
	At risk of malnutrition (MNA score 17–23.5)	265 (69.7)
	Satisfactory nutrition (MNA score ≥24)	99 (26.1)

Multimorbidity (two or more concurrent conditions) was present in 49.2% (95% CI: 44.2–54.2%; 187/380) of participants. Among those with comorbidities, one condition was reported by 33.4%, two by 30.3%, three by 12.6%, four by 5.8%, and five or six by 0.3% each (Figure 1).

**Figure 1:**
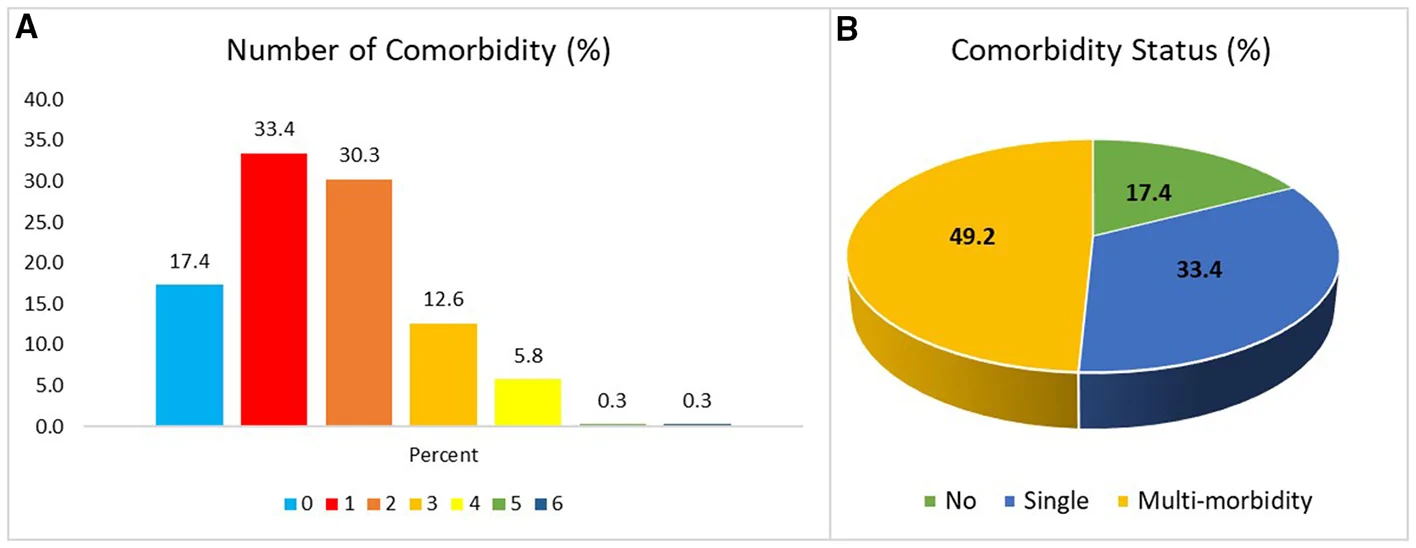
Distribution of the number of comorbidities and comorbidity status among the study participants (*n*= 380). **(A)** Distribution of participants according to the number of comorbidities. **(B)** Distribution of participants according to comorbidity status (no comorbidity, single comorbidity, and multimorbidity).

The descriptive statistics of quality of life (QOL) domains are presented in Table 2. The mean physical domain score was 22 ± 3, with a median of 23. The psychological domain had a mean score of 18 ± 3, while the social domain score was 8 ± 2. The environmental domain showed the highest mean score of 27 ± 5. The overall mean total QOL score was 80 ± 9, with a median of 80, indicating a moderate level of quality of life among the participants.

**Table 2:** Quality of life scores among the study participants.

Domain	Mean	SD	Median	25th Percentile	75th Percentile
Physical domain	22	3	23	21	24
Psychological domain	18	3	18	16	20
Social domain	8	2	8	6	10
Environment domain	27	5	27	23	30
Total QOL score	80	9	80	74	86

Spearman correlation analysis was performed to examine the relationship between the number of comorbidities, quality of life (QOL) domains, and nutritional status scores (Figure 2). The total QOL score showed strong positive correlations with the physical domain *(r* = *0.757, p* < *0.01)*, psychological domain *(r* = *0.734, p* < *0.01)*, social domain *(r* = *0.625, p* < *0.01)*, and environmental domain *(r* = *0.806, p* < *0.01)*. Significant moderate correlations were also observed between individual QOL domains, including the physical and psychological domains *(r* = *0.472, p* < *0.01)*, psychological and social domains *(r* = *0.453, p* < *0.01)*, and physical and environmental domains *(r* = *0.411, p* < *0.01)*.

**Figure 2:**
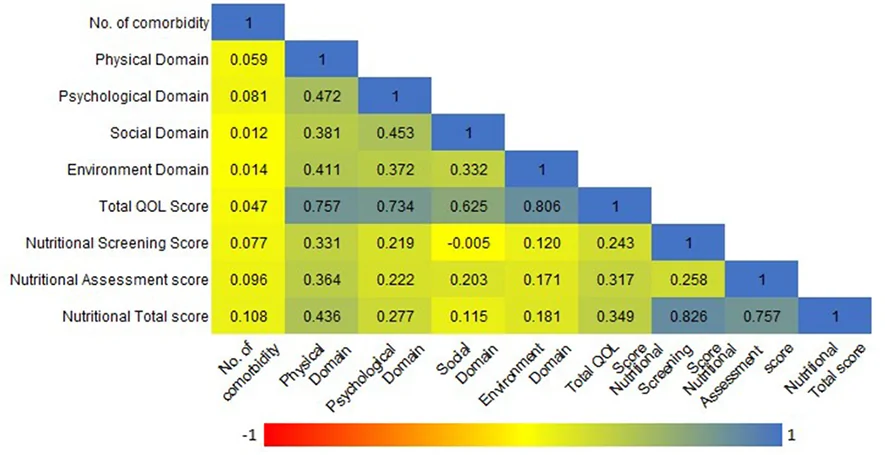
Spearman correlation heatmap showing relationships between comorbidity, quality of life domains, and nutritional status among study participants.

Regarding nutritional status, the total nutritional score showed strong positive correlations with the nutritional screening score *(r* = *0.826, p* < *0.01)* and nutritional assessment score *(r* = *0.757, p* < *0.01)*. Additionally, moderate positive correlations were observed between nutritional scores and QOL domains.

In contrast, the number of comorbidities showed weak and non-significant correlations with QOL domains and nutritional scores, suggesting that the raw number of chronic conditions may be a less sensitive indicator of health impact than their functional severity and individual burden on daily life; a distinction that warrants investigation using disease severity measures in future studies.

The association between socio-demographic characteristics and quality of life is presented in Table 3. Age showed a statistically significant association with quality of life (*p* < 0.001). Participants aged ≥70 years had a higher proportion with low QOL (68.7%) than those aged 60–69 years (46.7%). Educational status was also significantly associated with QOL (*p* = 0.011). A higher proportion of illiterate participants (66.7%) had low QOL compared with literate participants (51.0%). Nutritional status showed a significant association with quality of life (*p* = 0.014). Among those who were malnourished, 75.0% had low QOL, whereas participants with satisfactory nutrition were more likely to have better QOL (56.6%). However, gender, religion, occupation, monthly income, medical expenditure, sponsorship of medication expenditure, and presence of comorbidity were not significantly associated with quality of life.

**Table 3:** Association between socio-demographic factors, comorbidity, nutritional status, and quality of life among the study participants (*n* = 380).

Variable		Low QOL (≤ 80) ***n*** = **207**			Better QOL (>80) ***n*** = **173**			Chi-square/Exact test	*p*-value
		Count	Row %	Column %	Count	Row %	Column %		
Age (in Years)	60–69	115	46.7%	55.6%	131	53.3%	75.7%	16.790^a^	**<0.001**
	>=70	92	68.7%	44.4%	42	31.3%	24.3%		
Gender	Male	132	52.8%	63.8%	118	47.2%	68.2%	0.825^a^	0.364
	Female	75	57.7%	36.2%	55	42.3%	31.8%		
Religion	Hindu	202	54.3%	97.6%	170	45.7%	98.3%	0.212^b^	0.733
	Muslim	5	62.5%	2.4%	3	37.5%	1.7%		
Occupation	Unemployed	79	61.2%	38.2%	50	38.8%	28.9%	3.606^a^	**0.058**
	Employed	128	51.0%	61.8%	123	49.0%	71.1%		
Educational status	Literate	151	51.0%	72.9%	145	49.0%	83.8%	6.465^a^	**0.011**
	Illiterate	56	66.7%	27.1%	28	33.3%	16.2%		
Monthly income	Lower Class	128	55.4%	61.8%	103	44.6%	59.5%	3.971^c^	0.407
	Lower Middle Class	60	51.7%	29.0%	56	48.3%	32.4%		
	Middle Class	14	66.7%	6.8%	7	33.3%	4.0%		
	Upper Middle Class	1	20.0%	0.5%	4	80.0%	2.3%		
	Upper Class	4	57.1%	1.9%	3	42.9%	1.7%		
Monthly expenditure on medicine (Rupees)	Nil	61	54.0%	29.5%	52	46.0%	30.1%	3.011^c^	0.396
	<1,000	65	50.8%	31.4%	63	49.2%	36.4%		
	1,000–5,000	78	59.5%	37.7%	53	40.5%	30.6%		
	>5,000	3	37.5%	1.4%	5	62.5%	2.9%		
Sponsor of Medication expenditure	Nil	52	51.5%	25.1%	49	48.5%	28.3%	1.542^a^	0.462
	Own	83	52.9%	40.1%	74	47.1%	42.8%		
	Other	72	59.0%	34.8%	50	41.0%	28.9%		
Comorbidity	No	38	57.6%	18.4%	28	42.4%	16.2%	0.310^a^	0.578
	Yes	169	53.8%	81.6%	145	46.2%	83.8%		
MNA score	Malnourished (MNA score < 17)	12	75.0%	5.8%	4	25.0%	2.3%	8.472^a^	**0.014**
	At risk of malnutrition (MNA score 17–23.5)	152	57.4%	73.4%	113	42.6%	65.3%		
	Satisfactory nutrition (MNA score ≥24)	43	43.4%	20.8%	56	56.6%	32.4%		

^a^ Chi-square test, ^b^ Fisher's exact test, ^c^ Fisher-Freeman-Halton exact test. *p* < 0.05 considered statistically significant.

The association between socio-demographic characteristics and nutritional status is presented in Table 4. Age was significantly associated with nutritional status (*p* = 0.005), with a higher proportion of malnutrition observed among participants aged ≥70 years. Gender also showed a significant association (*p* = 0.002), with females accounting for 75.0% of the malnourished group, indicating a higher prevalence of malnutrition among older adults women. Occupation was significantly associated with nutritional status (*p* = 0.002), with unemployed participants more likely to be malnourished. Educational status demonstrated a strong association with nutritional status (*p* < 0.001). Illiterate participants were more likely to be malnourished compared to literate participants. Monthly income also showed a significant relationship with nutritional status (*p* = 0.019), with most malnourished individuals belonging to the lower socioeconomic class. Sponsorship of medication expenditure was marginally associated with nutritional status (*p* = 0.049). However, religion, medical expenditure, and comorbidity were not significantly associated with nutritional status.

**Table 4:** Distribution of nutritional status (MNA score) according to socio-demographic characteristics and comorbidity among the study participants (*n* = 380).

Variable		MNA score			Chi-square/Exact test	*p*-value
		Malnourished (score<17) *n* = 16	At risk of malnutrition (score 17–23.5) *n* = 265	Satisfactory nutrition (score ≥24) *n* = 99		
Age (in Years)	60–69	7 (43.8%)	163 (61.5%)	76 (76.8%)	10.573^a^	**0.005**
	>=70	9 (56.3%)	102 (38.5%)	23 (23.2%)		
Gender	Male	4 (25.0%)	180 (67.9%)	66 (66.6%)	12.398^a^	**0.002**
	Female	12 (75.0%)	85 (32.1%)	33 (33.3%)		
Religion	Hindu	16 (100.0%)	257 (97.0%)	99 (99.9%)	3.546^b^	0.112
	Muslim	0 (0.0%)	8 (3.0%)	0 (0.0%)		
Occupation	Unemployed	12 (75.0%)	85 (32.1%)	32 (32.3%)	12.556^a^	**0.002**
	Employed	4 (25.0%)	180 (67.9%)	67 (67.6%)		
Educational status	Literate	5 (31.3%)	210 (79.2%)	81 (81.8%)	21.383^a^	**< 0.001**
	Illiterate	11 (68.8%)	55 (20.8%)	18 (18.1%)		
Monthly income	Lower class	13 (81.3%)	155 (58.5%)	63 (63.6%)	10.993^c^	**0.019**
	Lower middle class	3 (18.8%)	91 (34.3%)	22 (22.2%)		
	Middle class	0 (0.0%)	13 (4.9%)	8 (8.8%)		
	Upper middle class	0 (0.0%)	4 (1.5%)	1 (1.1%)		
	Upper class	0 (0.0%)	2 (0.8%)	5 (5.5%)		
Monthly expenditure on medicine (Rupees)	Nil	7 (43.8%)	84 (31.7%)	22 (22.2%)	2.669^c^	0.447
	<1,000	5 (31.3%)	90 (34.0%)	33 (33.3%)		
	1,000–5,000	4 (25.0%)	85 (32.1%)	42 (42.4%)		
	>5,000	0 (0.0%)	6 (2.3%)	2 (2.2%)		
Sponsor of medication expenditure	Nil	7 (43.8%)	78 (29.4%)	16 (16.1%)	9.515^a^	**0.049**
	Own	4 (25.0%)	107 (40.4%)	46 (46.4%)		
	Other	5 (31.3%)	80 (30.2%)	37 (37.3%)		
Comorbidity	No	3 (18.8%)	48 (18.1%)	15 (15.1%)	0.463^a^	0.793
	Yes	13 (81.3%)	217 (81.9%)	84 (84.8%)		

^a^ Chi-square test, ^b^ Fisher's exact test, ^c^ Fisher-Freeman-Halton exact test. *p* < 0.05 considered statistically significant.

Multivariable logistic regression analysis in Table 5 showed that study participants aged ≥70 years had significantly higher odds of poor nutritional status compared with those aged 60–69 years (AOR = 2.16, 95% CI: 1.25–3.71, *p* = 0.006). Participants from the lower-middle socioeconomic group also had higher odds of poor nutritional status than those from the lower socioeconomic group (AOR = 2.18, 95% CI: 1.17–4.06; *p* = 0.014). Conversely, participants who financed their own medication expenditure (AOR = 0.45, 95% CI: 0.23–0.90; *p* = 0.023) and those whose medication expenses were supported by others (AOR = 0.49, 95% CI: 0.24–0.97; *p* = 0.041) had significantly lower odds of poor nutritional status than participants reporting no medication expenditure. Gender, occupation, educational status, and the remaining socioeconomic categories were not independently associated with poor nutritional status. The final model demonstrated adequate calibration according to the Hosmer–Lemeshow goodness-of-fit test (χ^2^ = 5.80, df = 8; *p* = 0.669). Multicollinearity was low, with all predictor Variance Inflation Factor values below 2.5 (maximum VIF = 2.31), indicating no problematic multicollinearity.

**Table 5:** Multivariable logistic regression analysis of factors associated with poor nutritional status among study participants (*n* = 380).

Variable	Adjusted odds ratio (AOR)	95% CI	*p*-value
Age ≥70 vs. 60–69 years	**2.16**	**1.25–3.73**	**0.005**
Female vs. Male	1.05	0.52–2.12	0.885
Employed vs. Unemployed	1.00	0.46–2.18	0.995
Illiterate vs. Literate	1.39	0.70–2.79	0.350
Lower-middle vs. Lower socioeconomic class	**2.18**	**1.17–4.06**	**0.014**
Middle vs. Lower socioeconomic class	0.97	0.36–2.61	0.950
Upper-middle vs. Lower socioeconomic class	1.52	0.16–14.42	0.714
Upper vs. Lower socioeconomic class	0.22	0.04–1.21	0.081
Own medication expenditure vs. Nil	**0.45**	**0.23–0.90**	**0.023**
Supported by others vs. Nil	**0.49**	**0.24–0.97**	**0.041**

AOR, adjusted odds ratio; CI, confidence interval.

**Model diagnostics:** Hosmer–Lemeshow goodness-of-fit test: χ^2^ = 5.80, df = 8, *p* = 0.669, indicating adequate model fit. Multicollinearity assessment showed no evidence of problematic collinearity (all VIF values < 2.5; maximum VIF = 2.31). *p* < 0.05 considered statistically significant.

## Discussion

The present study assessed the nutritional status, comorbidity profile, and quality of life among the older population residing in a rural community, and the findings provide crucial insights into the interrelationship between sociodemographic factors, health conditions, and wellbeing in advancing age. In the current study, two-thirds of participants (64.7%; *n* = 246) belonged to the 60–69-year age group, with a predominance of males, 250 (65.8%). The mean age of 71.23 ± 7.60 years observed in this study was higher than that reported by Devraj and D'mello (11) and Praveen et al. (12), where the mean age ranged between 65 and 66 years. Such differences may be due to varied sampling frames and demographic structures in rural vs. peri-urban settings, highlighting regional variation in older adults population profiles. The gender distribution showing more male participants aligns with the study of Piramanayagam et al. (13), who also noted a similar male preponderance among older adults respondents. Socioeconomically, most participants were classified as lower class according to the B.G. Prasad scale, consistent with national patterns observed in rural India. The current study's educational findings also echo the study by Abdu et al. (14), where a considerable proportion of older adults had low literacy levels, emphasizing persistent educational disadvantage in rural older adults groups. These sociodemographic patterns are important as they shape health-seeking behavior, accessibility to care, and vulnerability to nutritional and functional decline.

In terms of economic dependency, three-fourths, 279 (73.4%), of the study participants relied on family members for their medical expenses. This finding aligns with the HelpAge India survey by Dumka et al. (15), which reported that nearly half of older adults are financially dependent and often feel economically insecure. However, despite high access to health facilities, financial constraints remain a major barrier to availing care, a trend also reflected in our study, where dependency significantly influenced nutritional status and quality of life.

Physical functioning indicators showed that 318 (83.7%) of participants lived independently, which is encouraging for a rural older adults community. Moreover, nearly all (370; 97.4%) were able to self-feed, consistent with findings from Puducherry by Kalaiselvi et al. (16), suggesting that, despite chronic diseases, functional independence in basic activities may be preserved among many older adults. However, the present study found that only 134 (35.3%) had normal BMI, and a small proportion, 35 (9.2%), had low BMI, whereas the meta-analysis by Winter et al. (17) reported higher undernutrition rates globally. Importantly, age and nutritional status were significantly correlated, with older older adults more likely to be malnourished—a finding consistent with Krishnamoorthy et al. (18) and studies from Nepal and Tanzania (9, 19). Illiteracy and low socioeconomic status were major determinants of poor nutritional status, reinforcing the social gradient in health outcomes reported by Artacho et al. (20) and Aliabadi et al. (21). The present study further identified that the older adults who depended on themselves for medication expenses were significantly more likely to be malnourished, suggesting that financial burden directly influences dietary quality and health-seeking behavior. This difference may reflect dietary patterns, sampling differences, or cultural variations in food habits. Regarding dietary practices, most participants consumed three meals a day and included adequate protein in their diets. While this indicates awareness of balanced nutrition, 265 (69.7%) were at risk of malnutrition, and 16 (4.2%) were malnourished according to MNA classification. These findings are consistent with the studies by Amirkalali et al. (22) in Iran and Sharma et al. (9) in Nepal, who also reported that a considerable proportion of older adults individuals were at risk of malnutrition. The consistency of these results across countries highlights that malnutrition risk is a global concern among aging populations, especially in resource-limited settings. Anthropometric indicators such as Mid-Arm Circumference (MAC) and Calf Circumference (CC) were normal for more than 50% of the participants, which differs from findings of Bhagwasia et al. (23) who observed lower proportions of older adults with normal anthropometric measures. These discrepancies likely reflect differences in sampling strategies, reference populations, geographic ecology, and the sensitivity of anthropometric thresholds used across studies. Notably, Bhagwasia et al. (23) used a nationally representative longitudinal cohort, whereas the present study was restricted to a single rural field practice area, a difference that limits direct comparability. Nonetheless, the high proportion of the population at risk of malnutrition in our study underscores the urgent need for routine nutritional surveillance and targeted community-based nutritional interventions in rural Eastern India.

The comorbidity profile of the participants revealed chronic diseases in the study population, with nearly 315 (82.6%) participants experiencing at least one morbidity, which reduced QOL scores among the older adults. This was consistent with the studies conducted by Dasgupta et al. (24) and Thadathil et al. (25), in which the absence of comorbidity was found to be a determinant of better QOL scores. Some leading morbidities among the Indian population reported in previous studies included diabetes, chronic lung diseases, arthritis, and hypertension (26). A primary study conducted in rural Tamil Nadu outlined that cataract (57%) was the most common morbidity, followed by bone and joint disease (43.3%). Other morbidities were hypertension (14%), heart disease (9%), diabetes (8.1%), and asthma (6%) among older persons in rural areas (27). In the present study, most study participants had both hypertension (32.6%) and diabetes (24.2%). The above findings clearly indicate that these chronic morbidities are prevalent among the older adult(s), and primary care physicians should focus on these morbidities and provide comprehensive health services to the geriatric population. The predominance of hypertension and diabetes reflects common epidemiological trends in many adult populations, especially in settings with increasing lifestyle-related risk factors such as sedentary behavior, unhealthy diet, and aging demographics. The high prevalence of multi-morbidity 186 (49.2%) is particularly noteworthy, as co-existing chronic conditions are associated with greater healthcare utilization, reduced quality of life, and increased risk of complications. The coexistence of hypertension and diabetes is clinically important, as both conditions mutually aggravate cardiovascular and renal risks. A study showed that overall, 63% of older adults suffered from at least one non-communicable disease, and 30.7% of them had multimorbidity (28).

The relatively lower prevalence of lung disease and kidney disease may reflect the demographic characteristics or specific health profiles of the study group. However, the presence of osteoarthritis and anemia in notable proportions suggests additional burdens that may affect functional ability and overall wellbeing.

Overall, the data underscore the need for integrated chronic disease management, early screening, and lifestyle modification programs to mitigate the growing challenge of multi-morbidity. Tailored public health strategies focusing on prevention and control of hypertension and diabetes could significantly reduce the long-term disease burden in this population. Arthritis is the most prevalent condition, 45 (12%), followed by hypertension. Arthritis was also commonly reported in a study by Rent et al. (29) and others, indicating that degenerative joint diseases are a widespread concern among aging populations globally. The presence of comorbidities significantly curtailed daily functional activities, and participants with multiple conditions consistently reported lower QoL across domains. This is consistent with Dasgupta et al. (24) and Thadathil et al. (25), who identified freedom from comorbidity as a key determinant of better QoL. Importantly, the pathway from multimorbidity to poor QoL appears to be mediated through functional limitation, pain, and psychological distress rather than through comorbidity count alone, a mechanism supported by the weak direct correlation observed between comorbidity count and QoL scores in the present study. Integrated chronic disease management that addresses both physical and psychological dimensions of multimorbidity is therefore essential for improving QoL outcomes in this population.

Quality of life scores across physical, psychological, social, and environmental domains showed notable declines with increasing age and poorer nutritional status. Lower QOL scores were significantly associated with older age, illiteracy, and lower economic status. Similar patterns were observed in studies by Kumar et al. (30) and Rajini et al. (31), suggesting these determinants consistently affect QOL across different cultural contexts. Interestingly, some studies from developed nations, such as Low et al. (32), reported no association between age and QOL, highlighting the role of social security systems, healthcare access, and cultural norms in moderating aging experiences. Gender differences in QOL scores were also observed, where males generally scored higher in psychological and social domains, consistent with findings by Karmakar et al. (33) and Brajesh et al. (34). This could be attributed to greater social mobility, economic participation, and decision-making power historically afforded to men in rural settings. From a statistical perspective, adjusted odds ratios revealed that the older population aged ≥70 were significantly more likely to experience both malnutrition and comorbidity. Similar associations were echoed in the study by Singh et al. (35). Socioeconomic and educational disadvantages were significantly associated with malnutrition and comorbidities, supporting the evidence from Endale et al. (36) and Patel et al. (37). These relationships underscore the cumulative effect of lifelong social disadvantage on health in old age.

The findings clearly indicate that advancing age, socioeconomic deprivation, low education, and burden of comorbidities converge to adversely affect both nutritional status and QoL among rural older population in Eastern India. Malnutrition and multimorbidity are highly prevalent, and both were significantly associated with poor QOL outcomes. These results call for multilevel interventions focusing on nutritional support, early detection of chronic diseases, social support mechanisms, and geriatric-friendly health services. Strengthening government healthcare delivery and improving financial accessibility can significantly improve the health and wellbeing of rural older population.

## Limitations

One of the major limitations of the study is the use of a cross-sectional design where data are collected at a single point in time. Although this is an effective method for establishing an association between variables like nutrition, comorbidities, and quality of life (QoL) in older adults populations, it tends to restrict inferences regarding causality to some extent. The reliance on self-reported comorbidity data through the SCQ may introduce recall bias and reporting bias. The exclusion of bedridden or terminally ill older adults may lead to underestimation of the true burden of malnutrition and poor QoL in the community. Chronic illnesses may be underdiagnosed in rural settings with limited diagnostic infrastructure, potentially underestimating true morbidity. Further, important geriatric variables such as polypharmacy, functional disability (activities of daily living/instrumental activities of daily living; ADL/IADL), cognitive impairment, depression, social support, and detailed dietary intake patterns were not measured or controlled for, representing potential residual confounding. The dichotomisation of quality of life at the sample median, rather than at a validated clinical threshold, is a further limitation of this study. The study was conducted in a single rural field practice area of Eastern India, which limits the generalizability of findings to other geographic, cultural, or demographic contexts. Future studies that incorporate longitudinal designs and include comprehensive geriatric assessments are warranted to better characterize the complex interplay among nutrition, multimorbidity, and quality of life in older population.

## Conclusion

The present study emphasizes a large burden of malnutrition, comorbidities, and lower quality of life among older adults dwelling in rural Eastern India. Advancing age, female gender, illiteracy, low socioeconomic level, and lack of access to inexpensive healthcare were significantly associated with both malnutrition and multimorbidity. Higher comorbidity burden and nutritional deficiencies were substantially linked to lower QoL in a number of domains, highlighting how intertwined these issues are. These findings have direct implications for geriatric healthcare policy and rural health system strengthening. First, routine nutritional screening using the MNA should be integrated into primary healthcare for the older adults in rural settings. Second, multidisciplinary geriatric assessment and care should be planned, leveraging on the existing telemedicine set ups at primary and community health centers. Third, government programmes for the older adults, like the National Programme for Health Care of the Elderly (NPHCE) should be expanded and better supported to include nutritional supplementation, care for chronic diseases, and social support for financially dependent older adult(s). Fourth, health education campaigns targeting literacy, dietary practices, and medication adherence should be strengthened and scaled up in rural Eastern India. Future longitudinal research incorporating comprehensive geriatric assessments and controlling for confounders such as polypharmacy, depression, and functional disability is essential to establish causal pathways and inform evidence-based geriatric interventions.

## Data Availability

The raw data supporting the conclusions of this article will be made available by the authors, without undue reservation.
